# Delivering psychological and social support to children with cancer in India and their families: a position statement from the social and psychological taskforce of the Indian childhood cancer initiative

**DOI:** 10.3332/ecancer.2024.1812

**Published:** 2024-12-05

**Authors:** Rhea Daruvala, Ruchira Misra, Bindu Nair, Hiba Siddiqui, Usha Banerji, Valerie M Crabtree, Ramandeep Singh Arora

**Affiliations:** 1 Narayana Health, Bengaluru, Karnataka 560099, India; 2 MRRChidren’s Hospital, Thane, Maharashtra 400607, India; 3 Aroh, Coimbatore, Tamil Nadu 641006, India; 4 Max Institute of Cancer Care, Max Healthcare, New Delhi 110017, India; 5 Indian Institute of Technology, Hyderabad, Telangana, India; 6 St. Jude India Childcare Centers; 7 St. Jude Children’s Research Hospital in Memphis, Memphis, TN 38105, USA

**Keywords:** pediatric psycho oncology, childhood cancer, advocacy, low and middle income countries

## Abstract

The Indian childhood cancer initiative (ICCI) was established to address the critical gaps in childhood cancer care within India, where survival rates are significantly lower compared to high-income countries. While psychosocial support is a well-recognised component of comprehensive cancer care, its provision in India remains fragmented and inconsistent. This position statement, developed by the ICCI Psychological and Social Support Taskforce, outlines the urgent need for standardization of psychological and social care in pediatric oncology across the country. Informed by a thorough needs assessment and guided by international standards, this document proposes key areas of focus for integrating psychosocial support into pediatric cancer care. The taskforce identified eight priority areas essential for addressing the psychosocial needs of pediatric oncology patients and their families: ensuring holistic care through the integration of psychological and social support with medical treatment, early psychosocial assessment and intervention, interdisciplinary collaboration, community outreach, policy advocacy, research and the establishment of national standards for care. Early identification and intervention of psychosocial issues are critical to improving treatment adherence and overall patient outcomes. Furthermore, fostering interdisciplinary collaboration between medical professionals, psychologists and social workers is essential for delivering comprehensive care. The taskforce emphasises the importance of advocacy at both the policy level and community level, raising awareness about the psychosocial needs of children with cancer. The statement also highlights the necessity of expanding research in psychosocial oncology, particularly within the Indian context, to develop culturally sensitive interventions. Establishing national standards for psychosocial care will ensure equitable access to these services, addressing current disparities in service provision. In conclusion, this position statement advocates for systemic changes in pediatric cancer care, integrating psychosocial services into treatment protocols, fostering interdisciplinary collaboration and driving research and policy development to improve the quality of life for children with cancer and their families.

## Introduction to the position statement, methods and key areas for comprehensive psychosocial care in pediatric oncology

The World Health Organization estimates over 400,000 children are diagnosed with cancer each year [1]. While survival rates in high-income countries (HICs) are 80%–90%, they are much lower in low and middle-income countries (LMICs) like India and are estimated at 30%–40%. Factors such as access to care, education, early detection, treatment abandonment, financial burden and family support systems affect outcomes in LMIC. Effective cancer management, therefore, must include multi-modality treatments, medical supportive care and psychological and social support services to help patients cope [1].

The psychological impact of cancer is well documented. A cancer diagnosis can be emotionally and physically challenging for individuals and their families. Research illustrates that emotionally healthy patients cope better with their diagnosis and have a better long-term prognosis. Psychiatric conditions like depression and anxiety are associated with lower survival rates. Adequate support throughout the cancer journey, from prehabilitation to rehabilitation, is essential to ensuring emotional health and coping. This includes access to care, reliable information and knowledge about treatment needs and available support services. When children are diagnosed with cancer, the entire family system is said to be disrupted. Psychological support for the family aids in better treatment adjustment and coping, supporting the demands of intensive and lengthy cancer treatments.

In HIC, standards for social and psychological care in pediatric oncology are well established [2, 3]. However, in India, there are no agreed minimum standards or policies for the provision of such support. Pediatric psycho-oncology is a budding field with very few dedicated psycho-oncologists in pediatric oncology centers. Pediatric psycho-oncology in India remains underdeveloped, with fragmented services across the country. A few dedicated psycho-oncologists serve at major pediatric cancer centers, but formal psycho-oncology services remain scarce. Clinical services are mostly focused on large hospitals in metro areas, with limited reach to smaller towns. Educational programs for healthcare professionals in psycho-oncology are in their infancy, although some institutes have initiated short-term training programs. There is, therefore, an urgent need to expand psycho-oncology training, clinical services and research efforts. Social support is more available due to several not-for-profit organizations and social workers, but service provision is fragmented.

Recognising the emotional and social impact of cancer on young patients, the WHO Global Initiative for Childhood Cancer (WHO GICC) recommends psycho-oncology professionals and social workers support families during and after treatment [4]. An early response to the WHO GICC in India recognises the importance of social and psychological support [4]. The Indian childhood cancer initiative (ICCI) was

Launched in March 2023 to strengthen India’s childhood cancer control program, aiming for 60% survival and 100% access by 2030. To achieve this, ten task forces, including the ICCI Psychological and Social Support Taskforce, were created to standardise and streamline psychological and social support across India.

This task force addresses psycho-social challenges in pediatric oncology by integrating mental health professionals, social workers and community support into care. It raises awareness about the importance of psychological and social support within the medical community and advocates for policy changes to include these services. The task force consists of 36 members representing 14 hospitals and 11 civil society organizations and includes pediatric hemato-oncologists, social support staff, psychologists, psycho-oncologists, representatives from pediatric oncology organizations and nurses. The members of the taskforce also represent a wide range of geographical locations covering the expanse of the country and work in different settings including private hospitals, government hospitals, trust hospitals, civil societies, not for profit organizations.

The development of the areas to be addressed as part of the position statement was informed by a thorough needs assessment conducted across taskforce members representing various specialties, settings and stakeholders across the country to ensure a wide representation. This assessment involved gathering data from healthcare professionals, psycho-oncologists, social workers and families of children with cancer on key areas of psycho-social support that they felt were necessary to be addressed. This led to 31 areas being identified.

Following this, a prioritization and feasibility exercise was conducted to ensure that the proposed goals and interventions were practical and actionable. The task force used a multi-criteria decision-making process, where each recommendation was evaluated based on how important the area was to be implemented and the feasibility of implementation. This process allowed the task force to rank interventions in order of importance and ease of implementation, ensuring that high-priority and high-feasibility areas need could be addressed in the short term, while longer term goals were set for systemic changes.

Post this a smaller group of experts from the task force met online for several discussions about the areas. Some target areas were condensed or grouped together. The final eight areas in the position statement were then finalised based on discussion of the group and taking into consideration the Standards for psycho-social care for children with cancer and their families published in 2015 [5].

As a first step to advocating psycho-social care in pediatric oncology, the ICCI Psychological and Social Support Taskforce proposes a position statement emphasising the need for a comprehensive approach beyond medical treatment, advocating for a robust interdisciplinary support system for young cancer patients and their families in India.

## Every child with cancer should have holistic care

This includes ensuring the integration of psychological and social support seamlessly with medical treatment to provide holistic care for pediatric oncology patients and their families.

## Every child with cancer should have early assessment and intervention of their psycho-social needs

This includes implementation (and introduction where necessary) of proactive screening and assessment protocols to identify psycho-social needs early in the cancer care journey.

## Promotion of interdisciplinary collaboration is essential for delivery of social and psychological care to these children

Encouraging collaboration among medical professionals, psychologists, social workers and other experts is vital. This can be achieved by creating professional networks, mapping existing resources and developing cross-disciplinary training programs to enhance healthcare professionals’ skills and understanding.

## Encouraging community outreach and education to raise awareness at the community level about pediatric oncology and psychological and social aspects associated with childhood cancers

Raising community awareness about the importance of psychological and social support in pediatric oncology by conducting educational programs for families, caregivers and the public is essential to reduce stigma, enhance understanding and promote active participation in the support process. Establishing support groups for patients, caregivers and professionals can aid in outreach and education. Creating a database of relevant, reliable information on all aspects of cancer treatment, survivorship and psychological and social aspects for patients and caregivers to access.

## Advocate for policy to include psychological and social support services in pediatric oncology

Advocating for regional and national policy changes is necessary to include psychological and social support in standard pediatric oncology care while collaborating with healthcare policymakers to develop guidelines that integrate mental health and social services into treatment plans.

## Encourage research and innovation in psycho social aspects of pediatric oncology that are culturally sensitive and relevant

Promoting and conducting research on the psychological and social impacts of pediatric cancer, focusing on the Indian population will encourage innovation in support services through research-driven interventions to enhance the well-being of young cancer patients.

## All children with cancer should have access to psychological and social support services

Ensuring psychological and social support services are accessible to all children with cancer, regardless of socioeconomic factors by developing strategies to address disparities in access, ensuring vulnerable populations receive adequate support. This involves assessing the current situation in hospitals, identifying available support and addressing existing gaps.

## There should be standards for psychological and social support in pediatric oncology centers

Setting standards and protocols for various aspects of psychological and social support should include accreditation of professionals, requirements for formal training programs such as fellowships, creation of support groups and so on.

## Conclusion

As of now, psycho-social services in pediatric oncology remain inconsistently available. The lack of standardization in training and service provision limits access to comprehensive care. While a few centers provide psycho-social support, most children with cancer in India do not receive the holistic care needed to address their emotional and social needs. As a first step, the task force aims to establish national standards for the provision of psychological and social support in pediatric oncology centers, with the goal of achieving standardised and equitable access to these services across the country. In order to achieve this an initial guideline meeting was conducted in September 2024 with various stakeholders.

In summary, we advocate for a transformation in India’s pediatric cancer care, emphasising psychological and social support through interdisciplinary collaboration, community engagement, policy advocacy and research to enhance patients’ and families’ quality of life and care.

## Conflicts of interest

Rhea Daruvala, Ruchira Misra, Bindu Nair. Hiba Siddiqui, Usha Banerji, Ramandeep Arora, Valerie Crabtree – No conflict of interest.

## Funding

No funding to disclose.
